# Chlorpromazine as a Potential Antipsychotic Choice in COVID-19 Treatment

**DOI:** 10.3389/fpsyt.2020.612347

**Published:** 2020-12-23

**Authors:** Nemanja N. Muric, Nebojsa N. Arsenijevic, Milica M. Borovcanin

**Affiliations:** ^1^Department of Psychiatry, Faculty of Medical Sciences, University of Kragujevac, Kragujevac, Serbia; ^2^Psychiatric Clinic, Clinical Center Kragujevac, Kragujevac, Serbia; ^3^Center for Molecular Medicine and Stem Cell Research, Faculty of Medical Sciences, University of Kragujevac, Kragujevac, Serbia

**Keywords:** chlorpromazine, SARS-CoV-2, COVID-19, psychosis, infection

## Introduction

In the context of the ongoing pandemic of CoronaVirus Disease 2019 (COVID-19), caused by Severe Acute Respiratory Syndrome CoronaVirus-2 (SARS-CoV-2), there are already a lot of available research data considering the stressful impact of “social distancing”, but also difficulties that medical professionals experience, dealing with long shifts and frequent death witnessing, possible viruses exposure and above all stigmatization (1). Working at the University hospital requires that we also need to organize psychiatric consultations in the so-called “COVID-19 units” on a daily basis.

There are some recently published clinical experiences in managing symptoms of anxiety, depression, and psychosis in patients with diagnosed COVID-19 (2). Considering the urge of these newly established assignments for psychiatrists, but also the need to resolve psychotic symptoms in the best manner, we were wondering if antipsychotic treatment could accomplish positive effects, not only on psychotic symptoms but also on somatic state in patients with COVID-19. Therefore, we tried to find rational that the chlorpromazine (CPZ), a first-generation antipsychotic, could be useful in these purposes, considering its structure, and pharmacological origin, as well as anti-inflammatory potential and safety profile.

## SARS-CoV-2 Impact on Central Nervous System (CNS) and Behavior

The etiopathogenesis of this worldwide spread virus is still unknown, while somatic complications are various and proposed therapeutical protocols are inconsistent (3). Important for our scope of interest is that the SARS-CoV-2 penetration route into the brain is still the subject of various discussions. It has been proven that a virus enters the brain by migrating through the blood-brain barrier and interacts with the Angiotensin-Converting Enzyme 2 (ACE2) receptors, expressed by the brain tissue (4). Viral attachment to ACE2 receptors in the brain may cause arterial and venous thromboses, large-vessel ischemic strokes, intracerebral and subarachnoid hemorrhage widespread (5). There are three proposed potential ways in which the SARS-CoV-2 affects a person's behavior and mental functioning: above presented direct neuronal damage, but also by causing immune injury and hypoxia (5).

In a genetic review paper, Debnath et al. (6) tried to explain geographical discrepancies of SARS-CoV-2 by genetic variants of three gateways: ACE2, Human Leukocyte Antigen Locus, and Tool-like receptor and component pathways, leading to exaggerated immunity response. Recent studies pointed out that the activation of the immune-inflammatory pathways, as well as the occurrence of “cytokine storm” caused by COVID-19, plays an important role in the development of neuropsychiatric symptoms in affected patients, but also exacerbation of pre-existing symptoms among affected patients with a history of mental illnesses. Results based on computerized tomography and magnetic resonance imaging features presented by Poyiadji et al. (7) suggested the presence of COVID-19 associated acute necrotizing encephalopathy, indicating that it could be a potential consequence of a “cytokine storm” in the CNS of the patients with COVID-19. The fulminant and possibly fatal hypercytokinemia in hospitalized COVID-19 patients, presented as elevated blood plasma levels of interleukin (IL)-2, IL-7, granulocyte-colony stimulating factor, interferon-γ inducible protein 10, monocyte chemo-attractant protein 1, macrophage inflammatory protein 1-α, and Tumor Necrosis Factor-alpha (TNF-α) (8), and IL-6 and ferritin were much more expressed among fatal COVID-19 cases (9). SARS-CoV-2 attacks on hemoglobin, putting the lungs at the same time in a toxic and inflammatory state (10). It is well-known that serum levels of ferritin have been positively associated with inflammation, but opposite, hemoglobin showed a negative correlation (11).

These cytokine's perturbations could lead to behavioral changes, the clinical presentation of delirium, and psychotic symptoms. COVID-19 patients with no previous psychiatric history have developed psychotic symptoms, comorbid acute delirium, or psychosis conditions, characterized by thoughts of reference and structured delusional beliefs (12). The burden of long-term post-SARS-CoV-2 delirium may be significant, particularly for elderly patients who are more susceptible to post-infectious neurocognitive complications (13). Most of the cytokines involved in this “cytokine storm” have been previously linked with mental disorders, such as schizophrenia and bipolar disorder (14). And vice versa, influenza, SARS-CoV, and Middle East Respiratory Syndrome-related CoronaVirus (MERS-CoV) infections were shown to be associated with the onset of psychiatric symptoms (15, 16). Based on these understanding, neuropsychiatric consequences of “cytokine storm” seem highly likely in individuals with COVID-19 infection.

## Possible Advantages of Chlorpromazine Application in COVID-19 Induced Psychosis

Antipsychotics are widely used in the treatment of acute and chronic psychotic disorders, but they also showed to be effective in the treatment of agitated states in delirium and dementia, bipolar mania, and other psychopathological conditions (17). Also, there is a division of antipsychotics according to the receptor system on which they perform their action, so it could differ into serotonin-dopamine antagonists, dopamine antagonists, multireceptor antagonists, and partial dopamine agonists (18). Further, beyond the neurotransmitter's hypothesis, antipsychotics showed to have additional ability to attenuate type-2 immune response, similarly seen in asthma (19) and the question is what could be the rational choice for treating these complexly compromised COVID-19 patients when its somatic state is complicated with psychosis.

Chinese clinical recommendations were to use atypical antipsychotics olanzapine or quetiapine in patients with COVID-19 (20). Some antipsychotics showed to be more efficient in lowering these specific cytokines that were measured to be elevated in COVID-19 patient's serum, such as risperidone, olanzapine, and aripiprazole (21, 22), so maybe it could be more rational to use these antipsychotics in the first psychotic episode with possible secondary somatic benefits.

Interestingly, the search for an effective antimalarial drug led to the modification of phenothiazines and the consequent development of CPZ (23). CPZ belongs to the category of typical antipsychotics or neuroleptics, also known as first-generation antipsychotics, although the latest guidelines did not recommend it as a first line treatment. Furthermore, other indications for the use of this drug are in the treatment of nausea and vomiting (24), chronic hiccups (25), anxiety before surgery, acute intermittent porphyria, and tetanus symptoms (26). CPZ achieves his effects by the postsynaptic blockade at the D2 receptors in the mesolimbic pathway (to treat psychotic disorders) and combined blockade at histamine (H1), dopamine (D2), and muscarinic (M1) receptors in the vomiting center (antiemetic effect). CPZ has an excellent tolerance profile and it is easy to manage, but confirmed side effects are: sedation, dry mouth, hyperprolactinemia, and several endocrinal side effects, constipation and urinary retention, and in rare situations QT prolongation and potentially fatal malignant syndrome (26). On the other hand, apart from above-mentioned indications, where CPZ is widely used, less is known about its immunomodulatory and antiviral potential which may be useful during the COVID-19 pandemic.

## Immunomodulatory and Antiviral Properties of Chlorpromazine

Lately, more attention is focused on the immunomodulatory effects of CPZ which are accomplished by increasing the human blood levels of IgM (27). Along with that, CPZ reduces the serum levels of multiple pro-inflammatory cytokines such as IL-2, IL-4, and TNF, while boosting the anti-inflammatory chemical IL-10 in stimulated blood cells culture (27–30). Opposite to that, when different stimulants were used, Himmerich et al. (31) measured increased IL-4, IL-17, IL-2, and TNF-α levels, and Bertini et al. (32) suggested inhibitory effects of IL-1 *in vivo*. CPZ contributes to TNF production possibly due to more than one of its pharmacological activities and sufficiently protects against endotoxic shock simulated *in vitro* (28). Besides that, CPZ significantly increased C-reactive protein levels in the blood culture samples (33), reduces the secretion of IL-1β and IL-2 in mixed cultures of rat glial and microglia cells (34). Furthermore, it was point out that physiological importance of CPZ binding with hemoglobin in positive co-operative mode (35). CPZ can also inhibit phospholipase A2 and reduce the proinflammatory effects of platelet-activating factor, thromboxane (TxB2) (36) and leukotrienes (37), cascades that showed to be related into the progression of COVID-19 infection (38, 39).

Previously proven antiviral effects of CPZ indicate that its antiviral properties relevant to COVID-19 would be helpful. *In vitro*, antiviral properties of this molecule against various ribonucleic acid and deoxyribonucleic acid viruses were noted and can be very useful in the treatment of viral infections. CPZ has been reported to inhibit the replication of alphaviruses, hepatitis C virus, and coronaviruses: SARS-CoV, MERS-CoV, and Ebola virus (40–42).

An efficient target for CPZ antiviral action could be ACE2 receptors blockage in the brain (41). The usefulness of CPZ in the treatment of COVID-19 infection may be in its antiviral property due to the interaction with dynamin (cell membrane protein) to block clathrin-dependent endocytosis essential for coronavirus entry into the host cell. CPZ has a long half-life and it has a good safety profile at the doses required to treat numerous viral infections (43). CPZ biodistribution has a favorable antiviral profile: 20–200 times higher concentrations in the lungs that is important for the respiratory tropism of SARS-CoV-2, it is highly concentrated in the saliva for reduction of SARS-CoV-2 contagiousness and bypassing the blood-brain barrier could prevent neurological complications of COVID-19 (44). A search of the literature so far gives the impression that the adequate dose of this drug should be the subject of more detailed research in humans with COVID-19, but effective *in vitro* dosage for inhibiting viral replication of the MERS-CoV and SARS-CoV were non-toxic doses for cells (44, 45). Some studies suggested that CPZ inhibits the replication of MERS-CoV, implying that an effect on clathrin-mediated endocytosis is probably not the only antiviral mechanism. In the “battle” against both MERS-CoV and SARS-CoV, CPZ was one of the four The United States Food and Drug Administration approved compounds that were identified as an inhibitor of MERS and SARS in cell culture (46).

CPZ and chloroquine, drug actively used in COVID-19 treatment, have similarities in the structure-activity characteristics. Both drugs are clathrin-dependent endocytosis inhibitor, which is an important mechanism in the treatment of viral infections. *In vitro* study of Ferraris et al. (47) highlighted chloroquine as the main drug having the potential for drug repurposing, but also suggested the need for a chemical improvement of CPZ, because of its low selectivity index. Although CPZ was shown to be less efficient than chloroquine, it has much less pronounced adverse cardiological effects (48), and more prominent hepatotoxicity (49). Antipsychotics prescribed in COVID-19 could cause some adverse effects or interact with other drugs and possible interactions must be specially considered. In the simultaneous treatment of patients with antiviral medicines chloroquine and hydroxychloroquine, that showed shortening of the healing period and reduction of patient's infectiousness, adjustment of the previously or acutely administered psychopharmacotherapy is required. Interactions are possible at the level of amplification of dangerous side effects or effects on drug concentrations. In experimental models of malaria, it was shown that chlorpromazine was effective in potentiating chloroquine action (50). These previous findings could possibly give the rational explanation for the simultaneous application of chlorpromazine and chloroquine in the COVID-19. Even though, more research is needed on the effects of CPZ on the SARS-CoV-2 virus itself. We believe that this drug, because of its similarity to chloroquine, may be a good choice in the treatment of acute psychotic conditions in patients diagnosed with SARS-CoV-2 infection.

## Further Directions

The COVID-19 pandemic is still the major healthcare problem around the globe. Regardless of the current epidemiological situation, patients with psychiatric disorders are already highly vulnerable and could be in a specific risk of exposure to SARS-CoV-2. The neuropsychiatric manifestations could be a consequence of viremia and impact on CNS parenchyma, directly or through different systemic immunological disfunctions. Although CPZ is not always the first therapeutic choice in patients with an acute psychotic episode, considering its antimalaric origin and above-mentioned immunological effects, we wanted to remind on antiviral effects of this well-known antipsychotic that could find a place as a treatment option of mild or moderate COVID-19 cases. The aim of this paper was to draw attention to the antiviral effects of chlorpromazine and thus, to recommend administering this drug maybe as the first choice in an acute psychotic episode of patients with already diagnosed COVID-19, alone or in combination with hydroxychloroquine. Stip recently indicated (51) the necessity of differently designed clinical trials for off-label use of chlorpromazine in anti- COVID-19 treatment. We try to substantiate this approach, and although CPZ considers being obsolete and not regularly used as a first-line treatment of psychosis, we propose that its use should be reconsidered in the treatment of COVID-19 psychosis.

## Author Contributions

All authors were included in the designing of the manuscript, drafting the work, critical revision, final approval for all aspects of the work, and the final version to be published.

## Conflict of Interest

The authors declare that the research was conducted in the absence of any commercial or financial relationships that could be construed as a potential conflict of interest.
